# 

**Published:** 1892-07

**Authors:** 


### Notices of Dental Meetings.

### Pennsylvania and New Jersey State Societies.

A joint meeting of the Dental Societies of Pennsylvania and New Jersey will be held at Cresson Springs, Pa., beginning on Wednesday evening, July 20, 1892, and continuing for three days.

Papers and essays will be read by prominent members of the profession, and the clinics will be of the most varied character embracing every phase of prosthetic and mechanical dentistry.

It is the desire and expectation of the committee in charge of exhibits to have a very large display of instruments, appliances, etc., in fact, everything that relates to the workings of operative and mechanical dentistry. This exhibit will, without doubt, be the means of introducing many new and important features pertaining to the profession, which will be beneficial to all.

It is designed to make this meeting of the two societies one of most memorable ever held in the Middle States.

A cordial invitation is extended to all members of the profession to be present.

### National Association of Dental Faculties.

The ninth annual meeting of the National Association of Dental Faculties will be held at Niagara Falls, commencing on Monday, August 1st, 1892, at 10 o'clock A. M.

Each delegate must be a member of the faculty of the school he represents, and be provided with the proper credentials.

W. H. Eames, Pres.

J. D. Patterson, Sec'y.

### American Dental Association.

The thirty-second annual session of the American Dental Association will be held at Niagara Falls, N. Y., commencing at 10 o'clock A. M., Tuesday, August 2, 1892.

Geo. H. Cushing, Rec. Sec'y.

### South Carolina State Dental Association.

The Twenty-second Annual Meeting of the South Carolina State Dental Association will be held at Rock Hill, S. C., commencing on Tuesday, July 12th, 1892, at 10 A. M.

The Clinics will be a specially interesting feature of the meeting. A number of patients for these Clinics have been secured, and dental chairs will be provided.

A cordial invitation is extended to all members of the profession to attend this meeting.

Information regarding reduced rates on railroads, and at the hotels, will be furnished by the Committee on Arrangements.

The State Board of Dental Examiners will meet at the same time and place.

B. RUTLEDGE, D. D. S., Secretary.

### California State Dental Association.

THE twenty-third annual session of the California State Dental Association will be held in San Francisco, Cal., on Tuesday, July 19th, at 10 A. M., and continue four days.

It is expected that this will be a very interesting and instructive meeting. Members of the dental profession are cordially invited to attend.

THOMAS MORFFEW, President.

### National Association of Dental Examiners.

The annual meeting of the National Association of Dental Examiners will be held at Niagara Falls, Monday, August 1, 1892, at ten (10) A. M. All State Boards are invited.

FRED. A. LEVY, Sec'y.

THE WOMAN'S Pharmaceutical Association of Illinois is planning to conduct a model pharmacy in the Illinois building at the World's Fair.

### The Southern Dental Association.

The annual meeting of the Southern Dental Association will be held at Lookout Mountain, Chattanooga, beginning July 26th, and continuing four days.

The indications are that this will be one of the largest and most interesting meetings of this body ever held. The point is a central one; and also a very inviting one, no more attractive place could have been selected than Lookout Mountain. The temperature in summer is always pleasant, the atmosphere pure, and the scenery grand.

A most excellent programme has been prepared by the business committee for the occasion. The opportunity for good work by the Society is unprecedented, while the surroundings are of unmatched beauty, they are not of such a character as to draw the members away from the legitimate duties of the meeting; and the opportunities for social and professional intercourse will be all that could be desired. Ample provisions have been made for the comfort of the members and visiting dentists.

Arrangements have been made with all the railroads centering at Chattanooga for reduced rates, which will be upon the certificate plan. When purchasing a ticket at the starting point, procure a certificate upon which can be obtained return at one-third fare. Those going from Cincinnati and its neighboring towns will, of course, go via the Cincinnati Southern, this being the only direct road from Cincinnati to Chattanooga, and being one of the best roads of the country, and most perfectly equipped, makes a journey over it a real pleasure. It passes through the beautiful blue-grass region, and also through the Cumberland Mountains, the scenery of which must be seen to be appreciated. It is to be hoped that the journey over this road, and the attractions of Lookout Mountain, and the excellent meeting to be held, will be sufficient inducements for a large attendance from this part of the country. Trains over the Southern leave the Grand Central Station, Cincinnati, at 8 A. M., 11:30 A. M., and 8 P. M., each of which runs through in about twelve hours. We are assured that there will be a large attendance from all parts of the South.

At the same time and place the meeting of the General Executive Committee of the World's Columbian Dental Congress will be held. There will also be a meeting of several of the sub-committees, especially those of the Southern States, and altogether the occasion will be one of unusual interest and importance. It is to be hoped that all who can possibly do so will be present.

MEADVILLE, PA., May 5th, 1892.

At the 29th annual meeting of the Lake Erie Dental Association, which has closed to-day, the following resolution was unanimously adopted:

*Resolved*, That the manufacturers of porcelain teeth be requested to reduce the price of teeth in the same ratio to the decline in the price of platinum, that they made the advance on the price of teeth on account of the advanced price of platinum, and that we request the dental journals to publish this—*a true copy.*

C. D. ELLIOTT, Secretary.

### American Dental Association. Oh! for Niagara!

The regular annual meeting of the American Dental Association will be held at Niagara Falls, beginning Tuesday, August 2nd, and continuing four days.

This will be an occasion of very great interest to the profession generally, as many matters pertaining to the World's Columbian Dental Congress will be there considered, and perhaps call for important action. All the Sub-committees will be called together upon that occasion. The various State Committees will also be called together for consultation and devising plans of work.

The meeting of the National Association of Dental Faculties is also to be held at that time, and also the National Association of State Dental Examining Boards.

Railroads centering at Niagara will give, as we understand, reduced rates. Arrangements have been made for those going from or through Cincinnati, with Erie Lines, by which most excellent accommodations will be had. A Pullman buffet sleeper will be run through for the exclusive use of those going to the association, if a reasonable number will go. Very low rates are offered and a stop over at Chautauqua either going or coming, or both. A lake boat ride will be given all who desire it.

The time for returning can be arranged to suit those who desire to stop over.

This special car will leave Cincinnati C. H. & D. Station, Sunday, July 31st, at 5:55 p. m.

This is a delightful route, as all know who have traveled over the road.

Any further particulars desired, may be had by inquiry of the editor of the REGISTER, No. 122 West Seventh St., Cincinnati.

It is important that all who desire to go under this special arrangement should make it known to the editor of the REGISTER by July 25th.

### CONTENTS.

### THE DENTAL REGISTER,

A MONTHLY MAGAZINE DEVOTED TO THE INTERESTS OF THE

### DENTAL PROFESSION.

*Terms Reduced to* $2.00 *Per Year. Single Copies,* 25 *Cents.*

### EDITED BY PROF. J. TAFT, D.D.S.

—AND—

### Published by SAM'L A. CROCKER & CO., Ohio Dental and Surgical Depot.

The volume commences in January and closes in December.

The REGISTER being issued monthly is always fresh, therefore indispensable to the practitioner who desires to keep posted up with the new inventions and improvements which are daily developed. Neither expense nor effort will be spared to give value and variety to its contents. Articles will be illustrated with well executed engravings whenever they will add to the interest or assist to explanation.

Its extensive circulation and frequency of issue make it one of the BEST DENTAL ADVERTISING MEDIUMS in the United States.

All subscriptions must begin with January or July.

Local or special notices, five lines or less, will be inserted for $3 each insertion; over five lines, 50 cts. per line.

All advertising bills payable in advance, or at furthest, after the issue of the first number containing the advertisement. All transient advertisements to be paid for in advance.

☞CONTRIBUTIONS SOLICITED.

Exclusively Editorial Communications should be addressed to the Editor.

The latest number of the REGISTER may be ordered with or without other goods at any time, and all letters should be addressed to

SAM'L A. CROCKER & CO., Ohio Dental and Surgical Depot, Cincinnati, O.

